# Cockroaches of genus *Muzoa*: morphology of the male genital sclerites and description of one new species (Dictyoptera, Blattodea, Ectobiidae, Nyctiborinae)

**DOI:** 10.3897/zookeys.278.4603

**Published:** 2013-03-20

**Authors:** Andrés H. Vélez-Bravo

**Affiliations:** 1 Invertebrate Collection of the University of Puerto Rico at Mayagüez (UPRM-INVCOL), Mayagüez, Puerto Rico – Grupo de Entomología de la Universidad de Antioquia (GEUA), Medellín, Colombia

**Keywords:** Colombia, Dichotomous key, Male genital sclerites, *Muzoa curtalata* Vélez sp. n.

## Abstract

The male genital sclerites of cockroaches of genus *Muzoa* Hebard 1921 are described for first time and the new species *Muzoa curtalata* **sp. n.** is described and ilustrated. A dichotomous key to identify the species of genus *Muzoa* is given.

## Introduction

The genus *Muzoa* is distributed in Central and South America (Beccaloni 2007). It was erected by Hebard (1921) according to: 1) Interocular space wide; 2) Cerci broad and heavy, subspatulate with the apex rather sharp; 3) Subgenital plate symmetrical; 4) Cephalic femora with ventro-cephalic margins armed with minute and well spaced spiniform hairs and 5) Symmetrical tarsal claws. The species *Muzoa madida* Rehn, 1930 (Costa Rica) and *Muzoa simplex* Hebard, 1921 (Colombia) have been grouped based on these characters exclusively.

The detailed study of the genital sclerites of cockroaches performed by McKittrick (1964) significantly influenced the Blattodea systematics. From that moment, it was common to find descriptions of genital sclerites, mostly male, in works dedicated to the description of new species, since the descriptions prior to the 60’s were limited only to description the external morphology of the specimen, so that the genus *Muzoa* was not exception. Subsequently, new works appeared to supplement or correct the terminology proposed by McKittrick (Roth and Gurney 1969, Roth 1970, 1973) or in the worst case rejecting this and proposing a new terminology (Guthrie and Tindall 1968). Mizukubo and Hirashima (1987), Klass (1995) and Grandcolas (1996) studied the homologies of the different elements of the male genital sclerites. And so today, based on the detailed work developed by Klass (1995), the genital sclerites that were previously considered as a single unit is divided into several regions (e.g. genital sclerite R (right phallomere) composed of regions R1, R2,....R5), and these in turn are divided into subregions (e.g. R1d, R1v), thus modifying the initial nomenclature proposed by McKittrick.

Therefore, in this work the genus *Muzoa* is rediagnosed, now adding, morphological characters related to the male genital sclerites, and additionally, one new species is described for the genus.

## Methods

Observations of external morphological characters were made with Leica MS5 and MZ16 stereomicroscopes (magnification 10–64× and 7–115×), equipped with an ocular graticule for measurements of lengths and ratios. Drawings were prepared with Digital Camera Leica EC3 attached to the compound scope. Based on the digital image, illustrations were made with an illustration software, in order to highlight features of taxonomic significance. The methods for dissecting male genitalia followed Gutiérrez (2001). The descriptions are based on male specimens.

The morphological terminology followed Torre-Bueno (Nichols 1989). Specific structures such as wings, sutures of head and genital sclerites were described in accordance with Crampton (1925), Klass (1995) and Roth (2003). The term R3c is named here for first time referring to sclerotized region of the genital sclerite R (see Klass 1995 to recognize the other sclerotized regions). R3c refers to the lower right corner of the sclerotized region R3 which projects as a short or long arm depending on the species.

The descriptions of the male genital sclerites were made based on the permanent slides (belonging to the holotypes) and fresh material preserved in glycerine. Illustrations were made based on fresh material.

The insect Collection codens are in accordance with Evenhuis (2012), as follows:

**ANSP** USA, Pennsylvania, Philadelphia, Academy of Natural Sciences.

**CIB** Colombia, Medellín, Centro de Investigaciones Biológicas.

**MUJ** Colombia, Bogotá D.C., Pontificia Universidad Javeriana, Museo Javeriano de Historia Natural, Laboratorio de Entomología.

**UNAB** Colombia, Bogotá D.C., Museo Entomológico de la Facultad de Agronomía, Universidad Nacional de Colombia.

**USNM** USA, Washington D.C., National Museum of Natural History.

## Taxonomy

### Muzoa

Hebard, 1921

http://species-id.net/wiki/Muzoa

#### Dichotomous key to the species of *Muzoa*

**Table:** 

1	Cockroaches with tegmina and membranous wings well developed, completely covering the supra-anal plate (Figs 1, 2)	2
–	Cockroaches with tegmina and membranous wings shortened, reaching just the first abdominal tergite (Fig. 3). Lateral extension of L2a elongate and extended dorsally over the process “via” (Fig. 16); apex of the region R2 of the genital sclerite R (right phallomere), broad and truncated (Fig. 20)	*Muzoa curtalata* Vélez sp. n. (Colombia)
2	Head vertex convex (Fig. 4). Styli short, four times longer than wide. Process “via” slender, long and curved (Fig. 14). Lateral extension of L2a extending over the process “via” (Fig. 14).	*Muzoa simplex* (Colombia)
–	Head vertex straight (Fig. 5). Styli long, five times longer than wide (Fig. 12). Process “via” short, thick and straight (Fig. 15). Lateral extension of L2a not extending over the process “via” (Fig. 15)	*Muzoa madida* (Colombia, Costa Rica)

#### Remarks.

**Figures 1–6.**
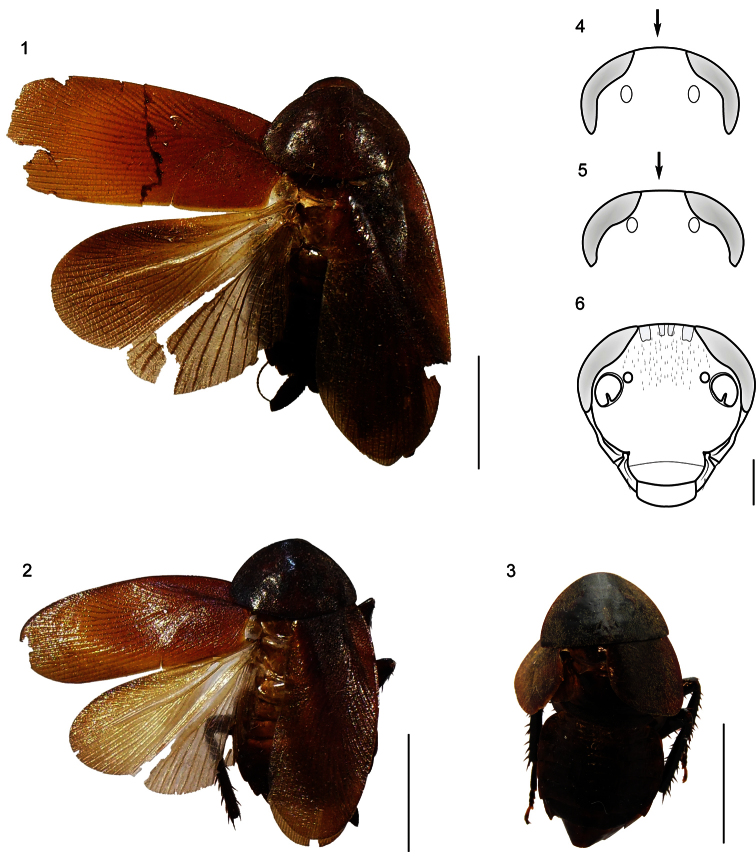
**1–3** Habitus (dorsal) of the species of genus *Muzoa*. **1** *Muzoa simplex* Hebard, 1921, holotype male (ANSP) **2** *Muzoa madida* Rehn, 1930, holotype male (ANSP). **3** *Muzoa curtalata* sp. n., holotype male (MUJ). Scale bar 1 cm. **4–6** Heads (ventral) of the species of genus *Muzoa* **4** *Muzoa simplex* **5** *Muzoa madida* **6** *Muzoa curtalata* sp. n. The arrow is indicating the shape of the vertex. Scale bar 1 mm.

Although the original descriptions of *Muzoa madida* (Fig. 2) and *Muzoa simplex* (Fig. 1) are extensive and detailed (see Hebard 1921 and Rehn 1930), these do not address the male genital sclerites, and in none other subsequent publication those structures have been described. Below are described and illustrated the male genital sclerites of these two species.

#### Redescription for the genus.

Species of medium size (24–27 mm male, 20–26 mm female), with body dark brown. The legs and antennae are entirely brown. Pronotum and tegmina reddish brown.

Head triangular and with big reniform eyes, extending antero-laterally beyond to the antennal socket, eyes are not globose; intraocular distance of the same length than distance between ocellar fenestra; face globose; gena and pleurostoma undivided, at least externally, so that the subgenal suture only present in the inner margin of pleurostoma; subantenal suture ending next to the inferior margin of the eye; the face and gena bare. Antennae filiform and setosas along their length; the first flagellar segment of the same length that the pedicel.

Pronotum parabolic, with its cephalic margin convex and the caudal margin truncated. In either sex, both pairs of wings are developed surpassing slightly the apex of the cercus, except in *Muzoa curtalata* sp. n. in which the males are brachypterous. Fore wings with the base of the remigium narrower than the base of vanal region (vannus) and the apex rounded; with discoidal sector longitudinal. Tegmina and pronotum densely covered with fine silky pubescence. Legs long and slender; cephalic coxa with a diagonal carina; antero-ventral margin of the front femur without spines heavy, only with short and heavy setae and with three terminal spines; postero-ventral margin of the hind femur with terminal spine; tarsomeres 1-4 with pulvilli, the first metatarsomere with its pulvilli covering only a 1/3 of its length; tarsal claws simple and symmetrical; arolium present.

**Figures 7–22.**
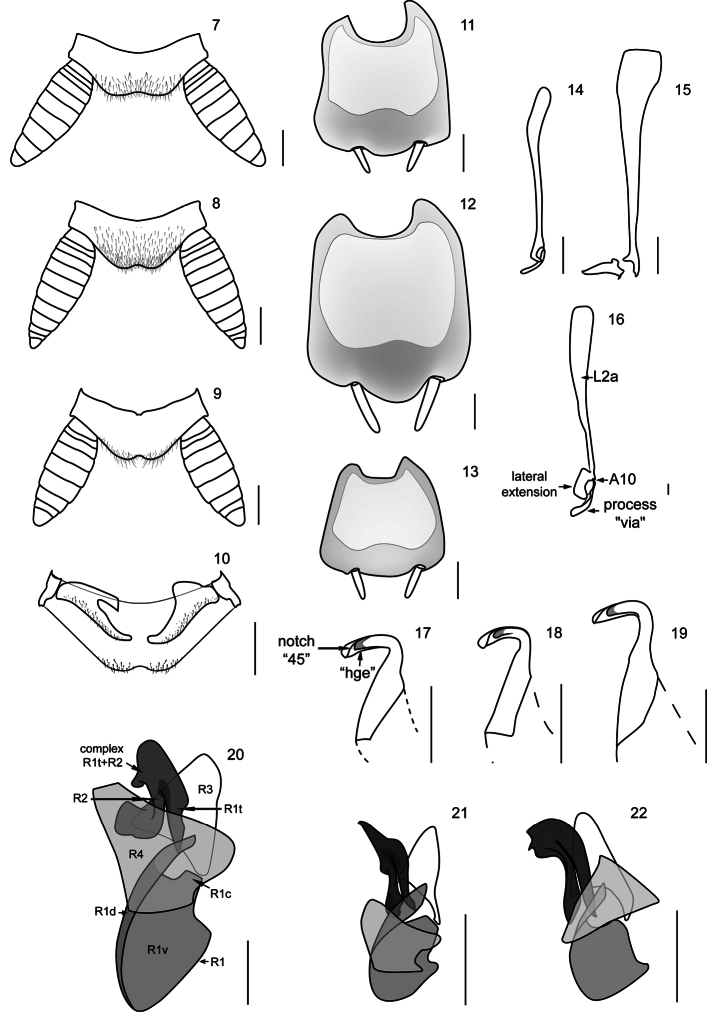
Supra-anal plate (dorsal), subgenital plate (vental), and male genital sclerites of the species of genus *Muzoa*. *Muzoa simplex* Hebard, 1921: **7** Supra-anal plate **11** Subgenital plate **14** Median sclerite L2 (dorsal) **18** Hook “hla” of L3 (ventral) **21** Right sclerite R (dorsal). *Muzoa madida* Rehn, 1930: **8** Supra-anal plate **12** Subgenital plate **15** Median sclerite L2 (dorsal) **19** Hook “hla” of L3 (ventral) **22** Right sclerite R (dorsal). *Muzoa curtalata* sp. n. (Holotype): **9** Supra-anal plate **10** Supra-anal plate (ventral) with the paraprocts **13** Subgenital plate **16** Median sclerite L2 (dorsal) **17** Hook “hla” of L3 (ventral) **20** Right sclerite R (dorsal) (sclerotized regions R1 [subregions R1c, R1d, R1v, R1t], R2, R3, R4). Scale bar 1 mm.

Abdomen often convex and short; first tergite unspecialized. Supra-anal plate tranverse and with the caudal margin produced and bilobed (Figs 7, 8). Cerci long, thick and subspatulate, composed of 9 to 12 segments; last segment small and narrower than the remaining segments. Ventrally, right paraproct specialized and transverse, this is articulated directly with the lateral margin of the supra-anal plate throughout its right lateral margin. Male subgenital plate symmetric, with styles similarly shaped (Figs 11, 12). Internally, attached to this plate, is located the membranous pouch with genital sclerites L2, L3, and R (right phallomere).

**Genital sclerites.**The male genital sclerites of the species of the genus *Muzoa* are formed by sclerites L2 (Figs 14, 15), L3 (Figs 18, 19), and R (right phallomere) (Figs 21, 22).

Genital sclerite L2 thin and elongated. Sclerotized region L2a and process “via” separated but closely articulated (Articulation 10 (A10), see Klass 1995) (Figs 14, 15). Process “via” finger-shaped, slender and elongate except for *Muzoa madida* in which it is shorter and thicker (Fig. 15). Region L2a is at least four times longer than process “via”. Whole region is slightly sclerotized.

Hook “hla” of sclerite L3 with the typical shape observed in most Ectobiidae and Blaberidae species, with distal area narrow and elongated; in addition to the notch “45”, the hook also exhibit groove “hge” along of its lower margin (Figs 17-19). In ventral view, basal area of hook “hla” longer than its apical area; left lateral margin of basal area, straight. Membranous tube of hook “hla” not covered by setae.

Genital sclerite R (right phallomere) formed by the sclerotized regions R1, R2, R3 and R4 (Figs 20–22). Region R1 as a large and bulky structure at the caudal region of sclerite R; subregion R1v broader than subregion R1d, which is a longitudinal narrow and elongated band, extending along the left lateral margin of R1; in species like *Muzoa madida* and *Muzoa simplex* this band does not reach the caudal margin of R1. Subregion R1d passing over the complex R1t+R2, surpassing its farthest right margin; its size varies among species. As in the other genera of Nyctiborinae, regions R1 and R3 articulated by the lower right corner of R3 and the upper right corner of R1. In all species of *Muzoa* the upper right corner of R1 (R1c) slightly projected (Figs 20–22). Subregion R1t is not fused with other areas of R1. Left arm of the complex R1t+R2 thicker than right arm, varying from apically rounded in *Muzoa simplex* to pointed in *Muzoa madida*. Complex R1t+R2 similar in size to region R3, located on its left margin, below the subregion R1d. Apex of R1t and R2 extending beyond caudal margin of R3.

Region R3 as a slightly sclerotized plate articulated by its lower right corner to R1c. This plate is longer than wide and its apex is rounded (Figs 20–22).

Region R4 as an elongated dorsal plate, covering R1 and R1t+R2 complex in part (Figs 20–22).

**Table 1.** Material examined.

**Species**	**Country**	**Locality**	**Method of collecting**	**Date**	**Collector’s names**	**Condition of specimen**	**Repository**
*Muzoa madida*	Colombia	Chocó, Nuquí, Jurubida, Emberá community. Edge of primary forest.	Shannon-165W	23 Oct. 1995	R. Vélez	Male. Pinned	CIB. Used to illustrate the genital sclerites
*Muzoa madida*	Costa Rica	Limón, forest near La Emilia. In dense second growth forest, 304 m.		15 Sep. 1937	Rehn	Male. Pinned	ANSP. Holtype 5481
*Muzoa madida*	Costa Rica	Turrialba. 900 m.			Heyne, Berlin-Wilm	Male. Pinned	USNM.
*Muzoa simplex*	Colombia	Boyacá, Muzo		Sep. 1919	A. María	Male. Pinned	ANSP. Holotype 9295
*Muzoa simplex*	Colombia	Chocó, Nuquí, Jurubida, Emberá community. Edge of primary forest	Shannon-165W	23 Oct. 1995	R. Vélez	2 Male. Pinned	CIB. Used to illustrate the genital sclerites
*Muzoa simplex*	Colombia	Cundinamarca, Bogotá		Jan. 1934	Guevara	2 Male.<br/> Pinned	USNM.
*Muzoa simplex*	Colombia	Cundinamarca, Tibacuy, Ins. Pol. Cumaca. 4°21'N, 74°27'W, 1647 m.		14 Nov. 1993	Valderrama	Male.<br/> Pinned	UNAB.

### Muzoa curtalata

Vélez sp. n.

urn:lsid:zoobank.org:act:0B136466-60A0-44D8-8DF8-528FDE38D010

http://species-id.net/wiki/Muzoa_curtalata

Figures 3, 6 9, 10, 13, 16, 17, 20

#### Type-locality.

Colombia, Valle del Cauca, Tuluá, Juan María Céspedes botanical garden, 4.029214, -76.160409, 1100 m, E. Amat leg. 24–31 Aug 1996.

#### Type-specimen.

Holotype male, pinned, with genitalia in a separate microvial. Original label: “Colombia. Valle. Mun. Tuluá. Jardín Botánico “Juan María Céspedes” 1100 m.s.n.m. E. Amat leg. 24–31 Ago 1996” MUJ – BLA - 025.

#### Differential diagnosis.

This species belongs to the genus *Muzoa* by: 1) Pronotum parabolic, with the caudal margin truncated; 2) antero-ventral margin of the cephalic femur without spines; 3) tarsal claws simple and symmetrical; 4) first abdominal tergite unspecialized; 5) supra-anal plate tranverse, with caudal margin produced and bilobed; 6) cerci long, thick and subspatulate; 7) male subgenital plate symmetric, with styles similarly shaped; 8) genital sclerites: process “via”, of the genital slcerite L2, finger-shaped and 9) hook “hla” of the genital sclerite L3, with groove “hge” along its lower margin. *Muzoa curtalata* differs from *Muzoa madida* and *Muzoa simplex* for its brachypterous condition. *Muzoa curtalata* is more closely related to *Muzoa simplex* for having a long lateral extension of L2a, which covers part of the process “via” (Figs 14, 16), different to *Muzoa madida*, in which the lateral extension is shorter and never covers part of the process “via” (Fig. 15).

#### Description.

Species of medium size (19.8 mm), with body dark brown; legs and antennae entirely brown. Pronotum and tegmina reddish brown (Fig. 3).

Head triangular and with big reniform eyes, extending antero-laterally beyond the antennal sockets; intraocular distance equal to distance between ocellar fenestra (1.3 mm) and lesser than distance between antennal sockets (2.0 mm) (Fig. 6); face globose; gena and pleurostoma undivided, at least externally, so that subgenal suture only present on the inner margin of pleurostoma; subantennal suture ending next to inferior margin of eye; face with many short setae on the frons, gena and remaining of face bare.

Pronotum parabolic, with cephalic margin convex and caudal margin truncated. Brachypterous. Fore wings coriaceous, lacking distinct veins; apex truncated, not surpassing the first abdominal tergite. Hind wings slightly developed, with reduced venation. Tegmina and pronotum covered densely with fine silky pubescence. Legs long and slender; cephalic coxa with a diagonal carina; antero-ventral margin of the front femur without spines heavy, only with short and thick setae, with three terminal spines; antero-ventral margin of middle and posterior femur with five and six spines correspondingly, postero-ventral margin with four and five spines respectively; tarsomeres 1-4 with pulvilli, first metatarsomere with its pulvilli covering only 1/3 of its length; tarsal claws simple and symmetrical; arolium present.

First abdominal tergite unspecialized. Supra-anal plate transverse, with caudal margin produced and bilobed (Fig. 9); cerci long, thick and subspatulate, composed of nine segments; last segment shorter and narrower than remaining segments (Fig. 9); ventrally, right paraproct transverse and claw-shaped (Fig. 10), articulated directly with the lateral margin of supra-anal plate through its right lateral margin. Subgenital plate symmetric, with styli similary shaped (Fig. 13). Internally, subject to this plate is located the membranous pouch with genital sclerites L2 (Fig. 16), L3 (Fig. 17), and R (right phallomere) (Fig. 20).

Genital sclerites. Genital sclerite L2 thin and elongated. Sclerotized region L2d and the process “via” separated but closely articulated (A10). Process “via” finger-shaped, slender and long (Fig. 16). Region L2a slightly sclerotized, at least four times length of “via”, with a lateral extension extending over the process “via”.

Hook “hla” of the genital sclerite L3 with distal area elongated; in addition to the notch “45”, with the groove “hge” along its lower margin (Fig. 17). Basal area of “hla” longer than its apical area, left lateral margin of basal area straight.

Genital sclerite R (right phallomere) formed by sclerotized regions R1, R2, R3 and R4 (Fig. 20). Region R1 as a large and bulky structure at the caudal region of sclerite R; subregion R1v much wider than subregion R1d, which is a narrow and elongated band, extending along left lateral margin of R1; subregion R1d projected over the apex of R1t (Fig. 20). R1c slightly projected, articulated to the lower right corner of R3 (Fig. 20). Subregion R1t is not fused with other areas of R1. Both arms of the complex R1t+R2 have more or less the same length. Left arm of the complex R1t+R2 thick, irregularly shaped, projected towards the left. Complex R1t+R2, similar in size to region R3, located on the left corner of the region R3, below the projection of R1d. Apex of R1t and R2 extended beyond the caudal margin of R3.

Region R3 as a nearly triangular, slightly sclerotized plate articulated by its lower right corner to R1c; apex of R3 rounded (Fig. 20).

Region R4 as a wide dorsal plate, covering R1 and R1t+R2 complex in part (Fig. 20).

Measurements(mm). Body length 19.8; pronotum maximum length × width 6.2 × 10.5; tegmen length × width 7.0 × 5.9; interocular width 1.3; interantennal sockets width 2.0.

#### Etymology.

*curtus* (L) = short, *alatus* (L) = winged. The name refers to the short tegmina of this species.

#### Distribution.

North of South America in the department of Valle del Cauca, Colombia.

## Supplementary Material

XML Treatment for Muzoa

XML Treatment for Muzoa curtalata
